# ATM/Chk2 and ATR/Chk1 Pathways Respond to DNA Damage Induced by Movento^®^ 240SC and Envidor^®^ 240SC Keto-Enol Insecticides in the Germarium of *Drosophila melanogaster*

**DOI:** 10.3390/toxics11090754

**Published:** 2023-09-06

**Authors:** Berenyce González-Marín, María Elena Calderón-Segura, Jeff Sekelsky

**Affiliations:** 1 Posgrado en Ciencias Biológicas, Universidad Nacional Autónoma de México, Posgrado en Ciencias Biológicas, Unidad de Posgrado, Edificio D, 1° Piso, Circuito de Posgrados, Ciudad Universitaria, Coyoacán, Ciudad de México 04510, Mexico; berenyce@ciencias.unam.mx; 2 Laboratorio de Toxicología Ambiental, Departamento de Ciencias Ambientales, Instituto de Ciencias de la Atmósfera y Cambio Climático, Universidad Nacional Autónoma de México, Ciudad Universitaria Coyoacán, Ciudad de México 04510, Mexico; 3 Department of Biology and Integrative Program for Biological and Genome Sciences, University of North Carolina at Chapel Hill, Chapel Hill, NC 27599, USA; sekelsky@unc.edu

**Keywords:** keto-enol insecticides, DNA damage response, *Drosophila germarium*

## Abstract

DNA damage response (DDR) pathways in keto-enol genotoxicity have not been characterized, and few studies have reported genotoxic effects in non-target organisms. The present study shows that concentrations of 11.2, 22.4, 37.3 mg/L of Movento^®^ 240SC and 12.3, 24.6, 41.1 mg/L of Envidor^®^ 240SC for 72 h oral exposure induced DSBs by significantly increasing the percentage of γH2AV expression in regions 2b and 3 from the germarium of wild type females of *Drosophila melanogaster* Oregon R, compared to the control group (0.0 mg/L of insecticides), via confocal immunofluorescence microscopy. The comparison between both insecticides’ reveals that only the Envidor^®^ 240SC induces concentration-dependent DNA damage, as well as structural changes in the germarium. We determined that the DDR induced by Movento^®^ 240SC depends on the activation of the *ATM^tefu^*, *Chk1^grp^* and *Chk2^lok^* kinases by significantly increasing the percentage of expression of γH2AV in regions 2b and 3 of the germarium, and that *ATR^mei−29D^* and *p53^dp53^* kinases only respond at the highest concentration of 37.3 mg/L of Movento^®^ 240SC. With the Envidor^®^ 240SC insecticide, we determined that the DDR depends on the activation of the *ATR^mei−29D^/Chk1^grp^* and *ATM^tefu^/Chk2^lok^* kinases, and *p53^dp53^* by significantly increasing the percentage of expression of γH2AV in the germarium.

## 1. Introduction

Pesticides are considered ubiquitous pollutants in the environment. Exposure to these compounds has been associated with alterations in genetic material and the development of various types of cancer [1,2,3]. Keto-enol insecticides are a new group of agrochemicals derived from tetronic and tetramic acids, which have been commercialized by Bayer since 2000 in Mexico [4,5]. These contain three active ingredients: Spirodiclofen (Envidor^®^ 240SC), Spiromesifen (Oberon^®^ 240SC) and Spirotetramat (Movento^®^ 240SC) [4,6]. They present a novel mechanism of action by interfering with lipid biosynthesis, acting as acetyl-coenzyme A carboxylase (ACCase) inhibitors [6,7,8]. Exposure to these insecticides has been associated with various toxic effects on non-target organisms. Spirodiclofen (Envidor^®^ 240SC) is classified as a possible carcinogenic agent by the International Agency for Research on Cancer (IARC, 2009); it induces uterine adenocarcinoma, Leydig cell hypertrophy, vacuolization, degeneration and hyperplasia of interstitial cells in mammalian testes [9,10]; it produces alterations during the embryonic development of zebrafish (*Danio rerio*) [11], oxidative stress, lipid peroxidation and DNA damage in *Allium cepa* meristems [12]. Spirotetramat (Movento^®^ 240SC) has been shown to significantly increase oxidative stress, and lipid peroxidation in amphibian *Bufo bufo gargarinzas* larvae [13], as well as DNA damage in earthworm coelomocytes (*Eisenia fetida*) [14]. Furthermore, it exerts teratogenic effects [11]; it is an endocrine disruptor [15]; and it affects lipid metabolism and causes mitochondrial lesions in zebrafish (*Danio rerio*) [11,16].

We recently reported that the Movento^®^ 240SC (Spirotetramat) and Envidor^®^ 240SC (Spirodiclofen) insecticides induce DNA damage in *Drosophila melanogaster* ovarian cells [17]. However, the molecular mechanisms of response to DNA damage induced by keto-enol insecticides have not been characterized.

The induction of DNA damage is considered one of the main risk factors for the development of genetic diseases, reproductive dysfunction, birth defects and carcinogenesis [18,19]. When DNA damage occurs, cells activate DNA damage response (DDR) mechanisms, which detect the site of the damage, amplifying a cascade of protein kinases and activation of downstream effectors that promote cell cycle arrest and DNA damage repair leading to apoptosis [20,21]. In the presence of DNA damage, an early event to DDR is phosphorylation of histone H2AX at carboxyl-terminal residue serine 139, known in mammals as γH2AX and in *D. melanogaster* as γH2AV [22,23,24]. The γH2AX variant is a very robust marker to detect double-strand breaks (DSBs), but also single-strand breaks (SSBs), DNA adducts, transcription blockade, and DNA replication [25,26]. γH2AX-producing lesions can be specifically immunodetected as discrete “foci” (sites) in interphase nuclei or mitotic chromosomes by specific fluorophore-labeled antibodies that recognize the phosphorylated residue of γH2AX, which is highly sensitive, allowing for the detection of γH2AX even when there are few DNA lesions [27]. These foci can be quantified by means of fluorescence microscopy directly as the number of positive cells or as number of foci per nucleus or indirectly through their size and intensity of fluorescence emitted [28,29,30]. This marker is dependent on the action of members of a family of kinases related to phosphatidylinositol 3-kinase (PI3K) which includes ATM (*ataxia-telangiectasia mutated* or Drosophila *telomere fusion* (Tefu)) and ATR in mammals (*related to ATM and Rad3* or *meiotic-41* in Drosophila (Mei-41)) [31,32]. Activated ATM/Tefu and ATR/Mei-41 phosphorylate several substrates, including Chk1/*Grapes* (Grp) and Chk2/*loki* (lok) kinases, which regulate cell cycle arrest, DNA repair, and apoptosis [33,34,35]. The ATR/Chk1 pathway is activated mainly in the presence of stalled replication forks and DNA single-strand breaks (SSBs), controlling cell cycle arrest and DNA repair in S and G2/M phases [32,36]. The ATM/Chk2 pathway is activated upon induction of DNA double-strand breaks (DSBs), regulating cell cycle arrest in the G1/S phase and the activity of the p53 tumor suppressor that promotes the expression of target genes associated with DNA repair and apoptosis [37,38]. In Drosophila, a single ortholog of p53 has been identified, compared to the three mammalian members (p53, p63 and p73), which has facilitated its study [39,40].

Drosophila is an excellent organism recommended by the European Center for the Validation of Alternative Methods (ECVAM) for research in toxicology [41] and used for the study of various human carcinogenic processes [42,43], including DNA damage response mechanisms in in vivo systems [44,45,46]. The Drosophila ovary has functioned as a system to characterize DNA damage response and repair mechanisms [47], specifically the germarium, the residence site of germinal stem cells (GSCs), the oocyte division, differentiation, and formation site [48], in which double-strand breaks (DSBs) are generated in a programmed manner during the meiotic recombination process [49]. A considerable number of gene-deficient mutants involved in DNA damage and repair response have been generated in Drosophila [50,51,52,53] and are used in the evaluation of the genotoxic and mutagenic potential of various chemical compounds, such as pesticides [54,55]. However, there are no studies on the response mechanisms to DNA damage induced by exposure to these agrochemicals, especially in the ovary germarium. Therefore, the present study is the first to report on the mechanisms of response to DNA damage (DDR) induced by exposure to the keto-enol insecticides Movento^®^ 240SC and Envidor^®^ 240SC using mutant strains of *D. melanogaster* (*ATM^tefu^*, *ATR^mei−29D^*, *Chk1^grp^*/*Chk2^lok^*, *Chk1^grp^*, *p53^dp53^*) and wild type (Oregon R) through γH2AV expression by confocal immunofluorescence microscopy.

## 2. Materials and Methods

### 2.1. Drosophila Melanogaster Strains

Table 1 describes the wild-type and DDR mutant strains of *Drosophila melanogaster* used in the present study.

### 2.2. Preparation of Keto-Enol Insecticide Yeast Paste

The keto-enol insecticides Movento^®^ 240SC (with Spirotetramat as active ingredient (cis-4-(ethoxycarbonyloxy)-8-methoxy-3-(2,5-xylyl)-1-azaspiro [4.5] dec-3-en-2-one)), registration number (RSCO-INAC-0103Z-301-409-015) and Envidor^®^ 240SC (with active ingredient Spirodiclofen (3-(2,4-dichlorophenyl)-2-oxo-1-oxaspiro [4.5] dic-3-en-4-yl 2,2-dimethylbutyrate)), registration number (RSCO-INAC-0103R-301-064-022) were donated by Bayer Crop Science Mexico (Mexico City, Mexico). Both insecticides were diluted with deionized water to the final concentrations of (11.2, 22.4, 37.3 mg/L) of Movento^®^ 240SC and (12.3, 24.6, 41.1 mg/L) of Envidor^®^ 240SC. Two hundred microliters of each concentration were mixed with yeast to form a paste that was deposited in the bottom of Drosophila Genesee Scientific ((Wilford, Nottingham, UK) food vials. The concentrations of both insecticides that induced DNA damage in ovarian cells were reported in a previous study and used in three independent experiments [17].

### 2.3. Treatment Scheme for Keto-Enol Insecticides Movento^®^ 240SC and Envidor^®^ 240SC

**Figure 1:**
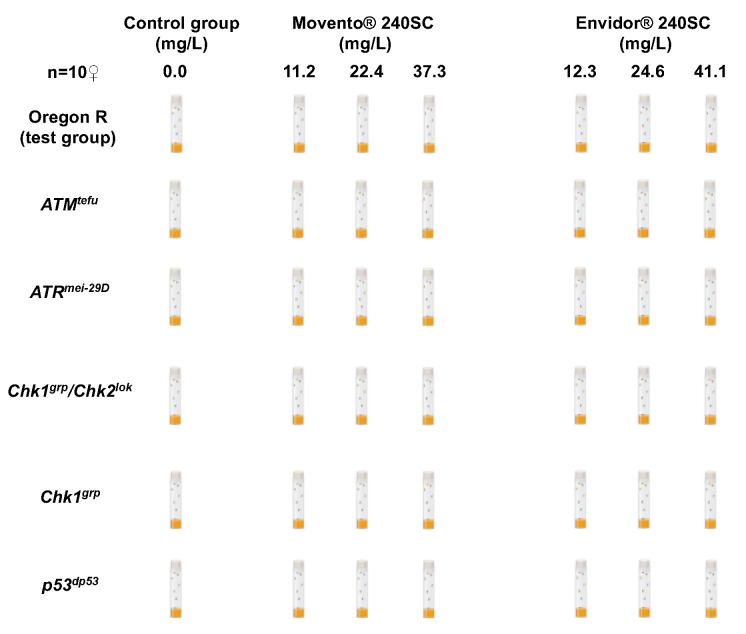
Treatment scheme for keto-enol insecticides Movento^®^ 240SC and Envidor^®^ 240SC in Oregon R wild type females and DDR mutants.

Forty female *Drosophila melanogaster* strains: Oregon R, *ATM^tefu^*, *ATR^mei−29D^*, *Chk1^grp^*/*Chk2^lok^*, *Chk1^grp^* and *p53^dp53^*, were collected for 3 days, grouped into 4 groups with 10 females each, to be incubated and fed in vials containing a yeast mixture with a concentration of 11.2, 22.4 y 37.3 mg/L of Movento^®^ 240SC and of 12.3, 24.6, 41.1 mg/L of Envidor^®^ 240SC, independently, for 72 h at 25 °C. As a control group, food without pesticide (0.0 mg/L) was used, under the same conditions as the experimental groups (Figure 1). Three independent experiments were performed.

### 2.4. Dissection of Ovaries

After 72 h of exposure to the keto-enol insecticides Movento^®^ 240SC and Envidor^®^ 240SC, the females of the strains, Oregon R, *ATM^tefu^*, *ATR^mei−29D^*, *Chk1^grp^*/*Chk2^lok^*, *Chk1^grp^* and *p53^dp53^*, from each experimental and control group were sacrificed for ovarian dissection. For each group, 20 ovaries (two per organism) were obtained, which were disaggregated into ovarioles (structural unit of the ovary). Ovarioles were fixed in 800 µL of fixative solution (165 µL of fresh 1X PBS, 600 µL of heptane, 25 µL of 16% formaldehyde and 10 µL of NP40), for 20 min. Subsequently, they were washed with 1 mL of 1X PBST (0.1% Tween-20 1X PBS), 3 times for 10 min.

### 2.5. Expression of γH2AV in the Ovary Germarium of Mutant and Wild-Type D. melanogaster Strains Exposed and Unexposed to Keto-Enol Insecticides by Confocal Immunofluorescence

The ovarioles of the wild-type and DDR mutant *D. melanogaster* strains after 72 h of exposure to the three concentrations of the keto-enol insecticides Movento^®^ 240SC and Envidor^®^ 240SC, and control groups, were incubated for 1 h in 1 mL of 1% PBST + BSA (10 mL PBST + 0.1 g BSA). After this time, they were incubated overnight in 500 μL of primary antibody diluted in 1 mL of (1% PBST + BSA) at 4 °C on a rocking nutator. Afterwards, they were washed three times with PBST and incubated in 500 μL of secondary antibody diluted 1:500 in (1% PBST + BSA) for 2 h, and for 10 min with 5 μL of DAPI. Subsequently, they were washed with 1X PBST three times for 15 min and mounted on a slide with 35 μL of ProLong Gold [49].

The following antibodies were used for staining the nuclei: primary (rabbit α-γH2AvD (p5137), 1:1000) (Rockland (Limerick, PA, USA)), secondary (Alexa fluor-488 goat α-rabbit IgG (H+L), 1:500) (ThermoFisher (Eugene, OR, USA)) and DAPI (4’,6-diamino-2 -phenylindole) (Sigma-Aldrich (Saint Louis, MO, USA)). The primary antibody γH2AvD was used as a marker for DNA double-strand breaks in the Drosophila germarium as described in [56].

### 2.6. Image Processing

Images of the germarium (apical region of the ovarioles) of females of the strain (Oregon R, *ATM^tefu^*, *ATR^mei−29D^*, *Chk1^grp^*/*Chk2^lok^*, *Chk1^grp^* and *p53^dp53^*) of each experimental and control group were taken with a Zeiss LSM880 confocal laser scanning microscope, using a 63 ×/0.65 NA oil immersion objective, using ZEN 2.1 software (Gottingen, Germany). The images were saved as .czi files with a key unknown to the reader for processing with FIJI software (Lousiana, NO, USA). The intensity of the emitted fluorescence of γH2AV was reported as the average of the total percentage of γH2AV expressed in the germarium modified from [57]. Finally, the most representative germarium images of each experimental and control group of each mutant and wild-type strain were selected; the images were cut out and figure panels were created in Adobe Photoshop CC 24.7 software.

### 2.7. Statistical Analysis

The values obtained for the percentage of expression of γH2AV in the germarium of DDR mutant and wild-type females were reported as means ± standard deviation of three independent experiments for each experimental and control group. The data were analyzed with two-way analysis of variance (ANOVA) and a multiple comparison post hoc test (Tukey) to determine significant differences between the experimental and control groups of each strain (*p <* 0.0001; *p* < 0.005); statistical analysis was performed using the GraphPad Prism version 9 program.

## 3. Results

### 3.1. Induction of DNA Damage in the Germarium in the Wild-Type Strain of Drosophila melanogaster (Oregon R) using Keto-Enol Insecticides Movento^®^ 240SC and Envidor^®^ 240SC

**Figure 2:**
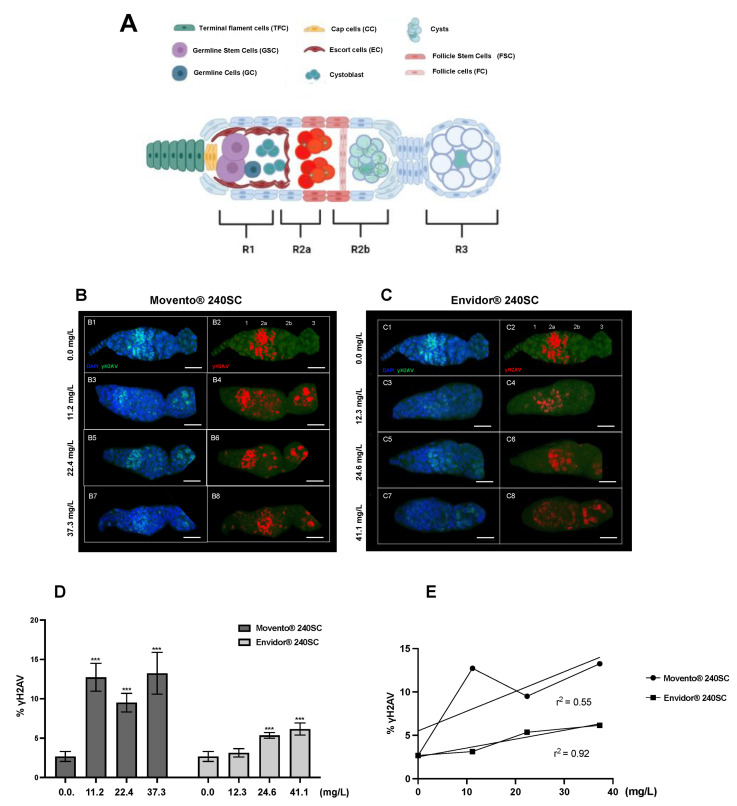
Germarium of wild-type Oregon R females immunostained against anti-γH2AV. (**A**) Schematic image of the germarium. Region 1: TFC, CC, GSCs, GC, EC, Cystoblast. Region 2a: Cysts, FSC. Region 2b: FC and Cyst oocyte. Region 3: FC, first ovarian chamber (first stage of oogenesis). (**B1**–**C8**) anti-γH2AV monoclonal antibody (green) to detect DSBs in DNA by immunofluorescence and DAPI for nuclei staining, red immunolocalization of γH2AV in the germarium, scale bar represents 10 μm. (**B1**,**B2**,**C1**,**C2**) Control group (0.0 mg/L), γH2AV (red) in region 2a of the germarium. (**B3**–**B8**) Germarium of females exposed to 11.2, 22.4, 37.3 mg/L of Movento^®^ 240SC, γH2AV (red) in regions 2b and 3. (**C3**–**C8**) Germarium of females exposed to 12.3, 24.6, 41.1 mg/L of Envidor^®^ 240SC. (**C3**,**C4**) γH2AV (red) in region 2a. (**C5**,**C8**) γH2AV (red) in regions 2b and 3. (**D**) Averages of three independent experiments of (%) expression of γH2AV in the germarium of the wild-type strain Oregon R with and without exposure to the keto-enol insecticides Movento^®^ 240SC and Envidor^®^ 240SC. *** Significant differences (*p <* 0.0001), two-way ANOVA and Tukey post hoc test. (**E**) Linear regression analysis.

Figure 2(B3–B8,D) shows the significant increase in DNA double-strand breaks and the percentage of γH2AV expression in regions 2b and 3 of the wild-type *Drosophila melanogaster* (Oregon R) germarium, after 72 h of oral exposure to the concentrations of 11.2, 22.4, and 37.3 mg/L of the keto-enol insecticide Movento^®^ 240SC, compared to the average value of basal damage in the DNA of the germarium of the control group (0.0 mg/L of pesticide) (*p <* 0.0001) (Figure 2(B1,B2,D)).

At a concentration of 12.3 mg/L of the insecticide Envidor^®^ 240SC, there were no significant differences in DNA damage and in the percentage of γH2AV expression, in relation to the average value of the control group (0.0 mg/L of pesticide) (*p <* 0.0001) (Figure 2(C1–C4,D)). However, at the concentrations of 24.6 and 41.1 mg/L of Envidor^®^ 240SC, we observed a significant increase in DSB in the DNA in regions 2b and 3 of the *D. melanogaster* (Oregon R) germarium (Figure 2(C5–C8)), and in the expression of γH2AV in relation to the control group (0.0 mg/L of pesticide) (*p <* 0.0001) (Figure 2D). Additionally, morphological alterations were determined in the germarium of *D. melanogaster* (Oregon R) exposed to the three concentrations of the insecticide Envidor^®^ 240SC (Figure 2(C3–C8)), compared to the morphology of the germarium of the control group (0.0 mg/L of pesticide) (Figure 2(C1,C2)).

The linear regression analysis of the means of γH2AV expression (DSB) induced by the keto-enol insecticides Movento^®^ 240SC and Envidor^®^ 240SC shows a concentration–effect response for Envidor^®^ 240SC (r^2^ = 0.92), but not for Movento^®^ 240SC (r^2^ = 0.55) (Figure 2E).

### 3.2. Response of ATM, Chk1 and Chk2 in the Germarium with DNA Damage Induced by the Keto-Enol Insecticide Movento^®^ 240SC

**Figure 3:**
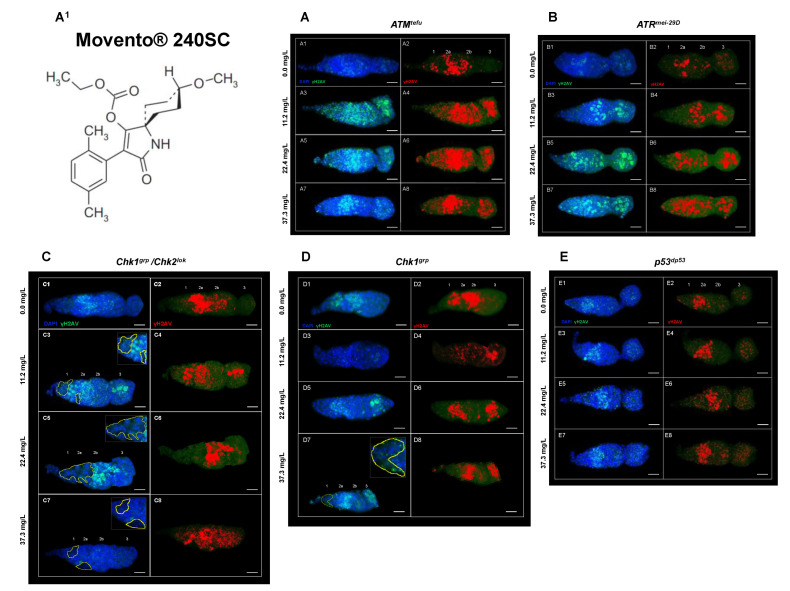
(**A**–**E**) Germarium of DDR mutant females immunostained against anti-γH2AV after 72 h of exposure to concentrations of 0.0, 11.2, 22.4, and 37.3 mg/L of Movento^®^ 240SC. Composite image in blue DAPI marking cell nuclei and in green γH2AV, red immunolocalization of γH2AV in the germarium, scale bar represents 10 μm. (**A^1^**) (a.i. Movento^®^ 240SC). (**A**) *ATM^tefu^*. (**A1**,**A2**) expression of γH2AV in regions 1, 2a, 2b of the germarium. (**A3**–**A8**) expression of γH2AV in all regions of the germarium and morphological alterations. (**B**) *ATR^mei−29D^.* (**B1**–**B4**) expression of γH2AV in regions 2a, 2b and 3 of the germarium. (**B5**–**B8**) expression of γH2AV in all regions of the germarium. (**C**) *Chk1^grp^*/*Chk2^lok^*. (**C1**,**C2**) expression of γH2AV in regions 2a and 2b of the germarium. (**C3**,**C4**) expression of γH2AV in regions 1, 2a and 3 of the germarium and absence of nuclei in regions 1 and 2a (yellow-dotted line). (**C5**,**C6**) expression of γH2AV in regions 2a and 2b of the germarium, absence of nuclei in regions 1 and 2a (yellow-dotted line) and morphological changes. (**C7**,**C8**) expression of γH2AV in all regions of the germarium, absence of nuclei in regions 1 and 2a (yellow-dotted line) and morphological changes. (**D**) *Chk1^grp^*. (**D1**,**D2**) expression of γH2AV in regions 1, 2a and 2b of the germarium. (**D3**,**D4**) expression of γH2AV in all regions of the germarium. (**D5**,**D6**) expression of γH2AV in regions 2a and 3 of the germarium. (**D7**,**D8**) expression of γH2AV in regions 2a, 2b and 3 of the germarium, absence of nuclei in region 1 (yellow-dotted line) and reduction in the size of the germarium. (**E**) *p53^dp53^*. (**E1**–**E4**) expression of γH2AV in regions 2a and 3 of the germarium. (**E5**–**E8**) expression of γH2AV in all regions of the germarium.

**Figure 4:**
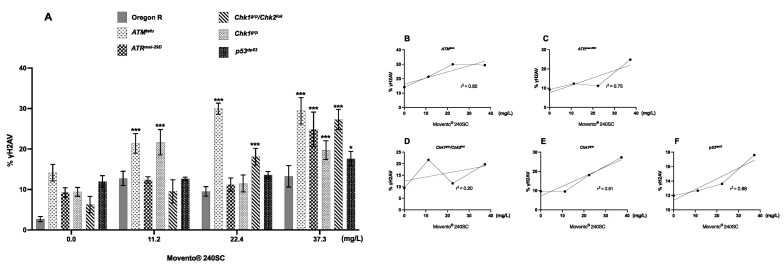
(**A**) Averages of three independent experiments of (%) expression of γH2AV in the germarium of the wild-type strain Oregon R and DDR mutants, with and without exposure to the keto-enol insecticide Movento^®^ 240SC. *** Significant differences (*p* < 0.0001), * significant differences (*p <* 0.005), two-way ANOVA and Tukey post hoc test. (**B**–**F**) Linear regression analysis.

Figure 3(A3–A8) shows evidence of DNA double-strand breaks (DSBs) in all regions of the germarium of *ATM^tef^*^u^ mutant females, after 72 h exposure to concentrations of 11.2, 22.4 and 37.3 mg/L of Movento^®^ 240SC (Figure 3(A^1^)), through the significant increase in the percentage of expression of γH2AV compared to the values of the control groups (*ATM^tefu^* 0.0 mg/L of pesticide) (Figure 3(A1,A2)), and wild-type strain (Oregon R) (exposed to the same concentrations of the insecticide) (*p <* 0.0001) (Figure 4A). Additionally, the three concentrations of Movento^®^ 240SC induced morphological alterations in the germarium (Figure 3(A3–A8)) compared to the morphology of the germarium of the control group (*ATM^tefu^* 0.0 mg/L of pesticide) (Figure 3(A1,A2)). The linear regression analysis shows a concentration–effect response in the *ATM^tefu^* strain (r^2^ = 0.82) (Figure 4B).

In *ATR^mei−29D^* mutant females, after 72 h of exposure to concentrations of 11.2, 22.4 and 37.3 mg/L of Movento^®^ 240SC, there was a significant increase in DSBs in DNA in regions 2b and 3 of the germarium (Figure 3(B3–B8)) and the percentage of expression of γH2AV compared to the values of the control group (*ATR^mei−29D^* 0.0 mg/L of pesticide) (*p <* 0.0001) (Figure 3(B1,B2)); however, at the concentrations of 11.2 and 22.4 mg/L, no significant differences were determined in the percentage of expression of γH2AV in the germarium, although at the concentration of 37.3 mg/L of Movento^®^ 240SC, we determined a significant increase in DSBs in the DNA and of the percentage of expression of γH2AV in the whole germarium (Figure 3(B7,B8)) in relation to the same experimental groups of the wild-type strain (Oregon R) (*p <* 0.0001) (Figure 4A). The linear regression analysis showed that the insecticide Movento^®^ 240SC does not induce concentration-dependent DNA damage in *D. melanogaster ATR^mei−29D^* (r^2^ = 0.75) (Figure 4C).

In *Chk1^grp^*/*Chk2^lok^* mutant females, after oral exposure to concentrations of 11.2 and 37.3 mg/L of Movento^®^ 240SC, we determined a significant increase in DSBs in DNA, in all regions of the germarium (Figure 3(C3,C4,C7,C8)) and the percentage of expression of γH2AV, compared to the control groups (*Chk1^grp^*/*Chk2^lok^* 0.0 mg/L of pesticide) (Figure 3(C1,C2)) and wild-type strain (Oregon R) (*p <* 0.0001) (Figure 4A). At a concentration of 22.4 mg/L of Movento^®^ 240SC, we did not determine any significant differences in the induction of DNA damage in germarium cells (Figure 3(C5,C6)) compared to the control groups (*Chk1^grp^*/*Chk2^lok^* 0.0 mg/L of pesticide) (Figure 3(C1,C2)) and wild-type strain (Oregon R) (*p <* 0.0001) (Figure 4A). However, exposure to the three concentrations of the insecticide Movento^®^ 240SC induce dead cell, evidenced by the absence of nuclei in regions 1 and 2a of the germarium (Figure 3(C3,C5,C7) yellow-dotted line); and at concentrations of 22.4 and 37.3 mg/L, it produces morphological changes in the germarium (Figure 3(C5–C8)) compared to the germarium of the control group (*Chk1^grp^*/*Chk2^lok^* 0.0 mg/L of pesticide) (Figure 3(C1,C2)). The linear regression analysis shows that the genotoxic effect of the insecticide Movento^®^ 240SC in *Chk1^grp^*/*Chk2^lok^* females is not concentration-dependent (r^2^ = 0.20) (Figure 4D).

In *Chk1^grp^* mutant females exposed for 72 h at a concentration of 11.2 mg/L, there was no significant increase in DSBs in the DNA and in the percentage of expression of γH2AV, in the germarium (Figure 3(D3,D4)) compared to the values for the control groups (*Chk1^grp^* 0.0 mg/L of pesticide) (Figure 3(D1,D2)) and wild-type strain (Oregon R) (*p <* 0.0001) (Figure 4A). However, at the concentrations of 22.4 and 37.3 mg/L of Movento^®^ 240SC, we observed a significant increase in DSBs in the DNA in regions 2a and 3 of the germarium (Figure 3(D5–D8)) and the percentage of expression of γH2AV compared to the values of the control groups (*Chk1^grp^* 0.0 mg/L of pesticide) (Figure 3(D1,D2)) and wild-type strain (Oregon R) (*p <* 0.0001) (Figure 4A). Additionally, at a concentration of 37.3 mg/L of Movento^®^ 240SC we observed inhibition of cell proliferation evidenced by the absence of cell nuclei in region 1 and reduction in the size of the germarium (Figure 3(D7) yellow-dotted line) compared to the control groups (*Chk1^grp^* 0.0 mg/L of pesticide) (Figure 3(D1,D2)). The linear regression analysis shows that the insecticide Movento^®^ 240SC has a concentration-dependent genotoxic response in *Chk1^grp^* females (r^2^ = 0.91) (Figure 4E).

Finally, in *p53^dp53^* mutant females, after exposure to concentrations of 11.2 and 22.4 mg/L of Movento^®^ 240SC, there was no significant increase in DNA double-strand breaks and in the percentage of expression of γH2AV, in the germarium (Figure 3(E3–E6)) compared to the values for the control groups (*p53 ^dp53^* 0.0 mg/L of pesticide) (Figure 3(E1,E2)) and wild-type (Oregon R) (Figure 4A). However, the concentration of 37.3 mg/L of Movento^®^ 240SC produced a significant increase in DNA DSBs in all regions of the germarium (Figure 3(E7,E8)) and the percentage of expression of γH2AV compared to the values of the control groups (*p53^dp53^* 0.0 mg/L of pesticide) (Figure 3(E1,E2)) and wild-type strain (Oregon R) (*p <* 0.005) (Figure 4A). The linear regression analysis shows that the genotoxic response of the insecticide Movento^®^ 240SC in *p53^dp53^* mutant females is not concentration-dependent (r^2^ = 0.88) (Figure 4F).

### 3.3. Response of ATM, ATR, Chk1, Chk1 and p53 in the Germarium of D. melanogaster with DNA Damage Induced by the Keto-Enol Insecticide Envidor^®^ 240 SC

**Figure 5:**
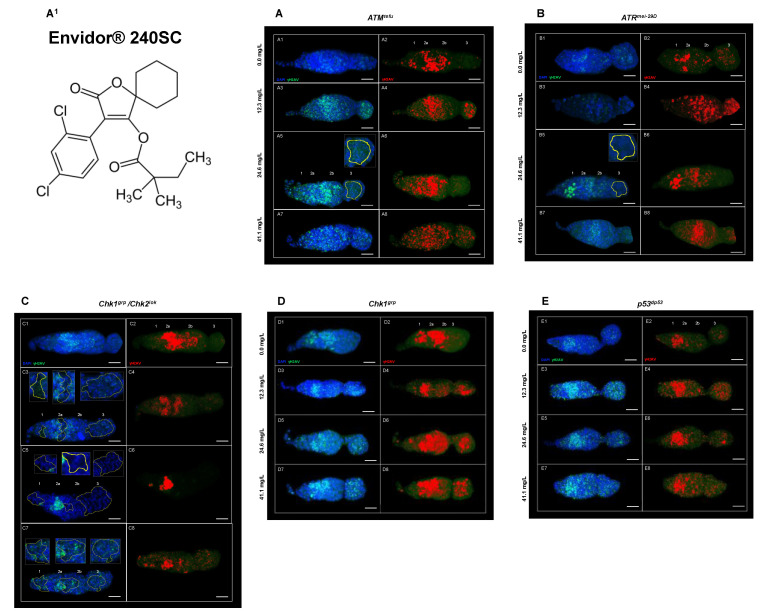
(**A**–**E**) Germarium of DDR mutant females immunostained against anti-γH2AV after 72 h of exposure to concentrations of 0.0, 12.3, 24.6, and 41.1 mg/L to Envidor^®^ 240SC. Composite image in blue DAPI marking cell nuclei and in green γH2AV, red immunolocalization of γH2AV in the germarium, scale bar represents 10 μm. (**A^1^**) Chemical structure of Spirodiclofen (a.i. Envidor^®^ 240SC). (**A**) *ATM^tefu^*. (**A1**,**A2**) expression of γH2AV in regions 1, 2a, 2b of the germarium. (**A3**,**A4**) expression of γH2AV in all regions of the germarium. (**A5**,**A6**) expression of γH2AV in regions 1, 2a and 2b of the germarium and absence of nuclei in region 3 (yellow-dotted line). (**A7**,**A8**) expression of γH2AV in all regions of the germarium. (**B**) *ATR^mei−29D^.* (**B1**–**B4**) expression of γH2AV in all regions of the germarium. (**B5**,**B6**) expression of γH2AV in regions 1, 2a and 2b of the germarium, absence of nuclei in region 3 (yellow-dotted line) and morphological changes. (**B7**,**B8**) expression of γH2AV in all regions of the germarium. (**C**) *Chk1^grp^*/*Chk2^lok^*. (**C1**,**C2**) expression of γH2AV in regions 2a and 2b of the germarium. (**C3**,**C4**) expression of γH2AV in regions 1 and 2a of the germarium and absence of nuclei in regions 1, 2a and 3 (yellow-dotted line). (**C5**,**C6**) expression of γH2AV in region 2a of the germarium, absence of nuclei in regions 1, 2a and 3 (yellow-dotted line) and morphological changes. (**C7**,**C8**) expression of γH2AV in all regions of the germarium and absence of nuclei in regions 1, 2b and 3 (yellow-dotted line). (**D**) *Chk1^grp^*. (**D1**,**D2**) expression of γH2AV in regions 1, 2a and 2b of the germarium. (**D3**–**D8**) expression of γH2AV in all regions of the germarium. (**E**) *p53^dp53^*. (**E1**,**E2**) expression of γH2AV in regions 2a and 3 of the germarium. (**E3**–**E6**) expression of γH2AV in all regions of the germarium. (**E7**,**E8**) expression of γH2AV in all regions of the germarium, morphological changes and reduction in the size of the germarium.

**Figure 6:**
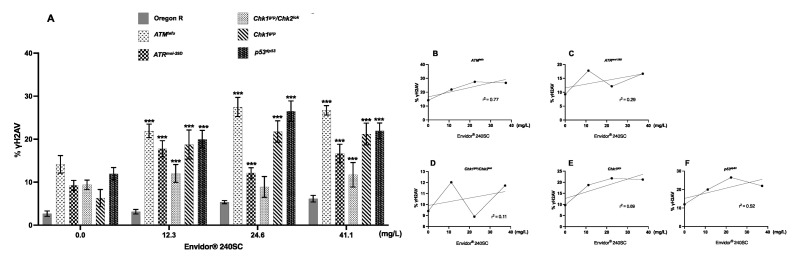
(**A**) Averages of three independent experiments of (%) expression of γH2AV in the germarium of the wild-type strain Oregon R and DDR mutants, with and without exposure to the keto-enol insecticide Envidor^®^ 240SC. *** Significant differences (*p <* 0.0001), two-way ANOVA and Tukey post hoc test. (**B**–**F**) Linear regression analysis.

Figure 5(A3–A8) show a significant increase in DNA DSBs in all regions of the germarium of *ATM^tefu^* mutant females after 72 h exposure to concentrations of 12.3, 24.6 and 41.1 mg/L of Envidor^®^ 240SC (Figure 5(A^1^)) and the percentage of expression of γH2AV compared to the control groups (*ATM^tefu^* 0.0 mg/L of pesticide) (Figure 5(A1,A2)) and wild-type strain (Oregon R) (*p <* 0.0001) (Figure 6A). Additionally, at a concentration of 24.6 mg/L of Envidor^®^ 240SC, we observed the absence of nuclei in region 3 (Figure 5(A5) yellow-dotted line) compared to the control group (*ATM^tefu^* 0.0 mg/L of pesticide) (Figure 5(A1,A2)). The linear regression analysis shows that the genotoxic response of the insecticide Envidor^®^ 240SC in *ATM^tefu^* females is not concentration-dependent (r^2^ = 0.77) (Figure 6B).

In *ATR^mei−29D^* mutant females, after 72 h of exposure to concentrations of 12.3, 24.6 and 41.1 mg/L of Envidor^®^ 240SC, there was a significant increase in DSBs in DNA of the germarium (Figure 5(B3–B8)) and the percentage of expression of γH2AV compared to the control groups (*ATR^mei−29D^* 0.0 mg/L of pesticide) (Figure 5(B1,B2)) and wild-type strain (Oregon R) (*p <* 0.0001) (Figure 6A). At a concentration of 24.6 mg/L of Envidor^®^ 240SC, we observed the absence of nuclei in region 3 (Figure 5(B5) yellow-dotted line) compared to the control group (*ATR^mei−29D^* 0.0 mg/L of pesticide) (Figure 5(B1,B2)). The linear regression analysis shows that the genotoxic response of the insecticide Envidor^®^ 240SC in *ATR^mei−29D^* mutant females is not concentration-dependent (r^2^ = 0.29) (Figure 6C).

In Drosophila *Chk1^grp^*/*Chk2^lok^* females exposed to 12.3 and 41.1 mg/L concentrations of Envidor^®^ 240SC for 72 h, there is a significant increase in DNA double-strand breaks in regions 1 and 3 of the germarium (Figure 5(C3,C4,C7,C8)) and in the percentage of expression of γH2AV, compared to control groups (*Chk1^grp^*/*Chk2^lok^* 0.0 mg/L of pesticide) (Figure 5(C1,C2)) and wild-type strain (Oregon R) (*p <* 0.0001) (Figure 6A). At a concentration of 24.6 mg/L of Envidor^®^ 240SC, there was no increase in DSBs in the DNA and in the percentage of expression of γH2AV in the germarium (Figure 5(C5,C6)) compared to the control groups (*Chk1^grp^*/*Chk2^lok^* 0.0 mg/L of pesticide) (Figure 5(C1,C2)) and wild-type strain (Oregon R) (*p <* 0.0001) (Figure 6A). However, exposure to the three concentrations of the insecticide Envidor^®^ 240SC inhibits cell proliferation, as evidenced by the absence of nuclei in all regions of the germarium and the morphological changes (Figure 5(C3,C5,C7) yellow-dotted line) compared to the control group (*Chk1^grp^*/*Chk2^lok^* 0.0 mg/L of pesticide) (Figure 5(C1,C2)). The linear regression analysis shows that there is no concentration–effect response for the insecticide Envidor^®^ 240SC in *Chk1^grp^*/*Chk2^lok^* females (r^2^ = 0.11) (Figure 6D).

In *Chk1^grp^* mutant females, exposure to concentrations of 12.3, 24.6 and 41.1 mg/L of Envidor^®^ 240SC significantly increases DSBs in DNA in all regions of the germarium (Figure 5(D3–D8)) as well as the percentage of expression of γH2AV, compared to the control groups (*Chk1^grp^* 0.0 mg/L of pesticide) (Figure 5(D1,D2)) and wild-type strain (Oregon R) (*p <* 0.0001) (Figure 6A). The linear regression analysis shows that there is no concentration–effect response for the insecticide Envidor^®^ 240SC in *Chk1^grp^* females (r^2^ = 0.69) (Figure 6E).

Finally, in *p53^dp53^* mutant females of *D. melanogaster*, exposure to concentrations of 12.3, 24.6 and 41.1 mg/L of Envidor^®^ 240SC significantly increased DSBs in DNA in all regions of the germarium (Figure 5(E3–E8)) as well as the percentage of expression of γH2AV compared to the control groups (*p53^dp53^* 0.0 mg/L of pesticide) (Figure 5(E1,E2)) and wild-type strain (Oregon R) (*p <* 0.0001) (Figure 6A). Concentrations of 24.6 and 41.1 mg/L produced morphological alterations and a reduction in the size of the germarium (Figure 5(E5–E8)) compared to the control groups (*p53^dp53^* 0.0 mg/L of pesticide) (Figure 5(E1,E2)). The linear regression analysis shows that there is no concentration–effect response for the insecticide Envidor^®^ 240SC in the *p53^dp53^* mutant (r^2^ = 0.52) (Figure 6F).

## 4. Discussion

The commercial keto-enol insecticides Movento^®^ 240SC and Envidor^®^ 240SC and their active ingredients (Spirotetramat and Spirodiclofen) have been shown to induce DNA damage in non-target organisms [12,14]. In a previous study, we reported that the commercial acaricides Movento^®^ 240SC and Envidor^®^ 240SC induce a significant increase in damage in the ovary DNA of wild-type *Drosophila melanogaster* (Oregon R) [17]. However, there were no reports on DNA damage response (DDR) pathways. The present study used the expression of histone γH2AV as a marker of DNA double-strand breaks (DSBs) in the ovarian germarium of *Drosophila melanogaster* in the wild-type strain Oregon R and DDR mutants: *ATM^tefu^*, *ATR^mei−29D^*, *Chk1^grp^*/*Chk2^lok^*, *Chk1^grp^* and *p53^dp53^* exposed for 72 h orally to elucidate DDR pathways.

The results of the expression of histone γH2AV in the germarium of the ovaries of wild-type *D. melanogaster* (Oregon R) after exposure to concentrations of 11.2, 22.4, 37.3 mg/L of Movento^®^ 240SC and 24.6, 41.1 mg/L of Envidor^®^ 240SC for 72 h show a significant increase in DNA double-strand breaks (DSBs) in regions 2b and 3 of the germarium. However, at a concentration of 12.3 mg/L of Envidor^®^ 240SC, there were no significant changes in DSB production in the germarium of *D. melanogaster* (Oregon R) compared to the percentage of endogenous expression in region 2a of the control group. (0.0 mg/L) (*p <* 0.0001). However, exposure to the three concentrations of Envidor^®^ 240SC produced structural changes in the germarium, compared to the germarium of the control group.

It has been reported that the endogenous induction of DSBs for meiotic recombination and visualization through the expression of γH2AV in *Drosophila melanogaster* is exclusively found in oocytes in the pachytene subphase of prophase I of meiosis I, in region 2a. These are repaired before differentiation into region 3 [47,58]. Therefore, the expression of γH2AV in regions 2b and 3 of the germarium after exposure to Movento^®^ 240SC and Envidor^®^ 240SC, indicate that both insecticides act as genotoxic agents through DSB induction. When comparing the induction of DNA damage in the germinal cells of the germarium in relation to both insecticides, we observed that only the insecticide Envidor^®^ 240SC induces concentration-dependent DNA damage, as well as structural changes of the germarium in wild-type Oregon R females. It is reported that one of the mechanisms of genotoxicity and cytotoxicity of Spirodiclofen (active ingredient of Envidor^®^ 240SC) is the induction of oxidative stress by increasing the production of reactive oxygen species (ROS), such as superoxide anion (O2−•), the hydroxyl radical (HO•), hydrogen peroxide (H_2_O_2_), with increased activity of endogenous antioxidants SOD and CAT, and induction of lipid peroxidation [12]. One of the effects of lipid peroxidation is the alteration of the assembly, composition, and permeability of cell membranes, altering their structure and function [59], which might justify the induction of ultrastructural alterations in the germarium after exposure to Envidor^®^ 240SC. In female zebrafish (*Danio rerio*), an induction of morphological alterations in the ovary, oxidative stress and lipid peroxidation were reported after exposure to the keto-enol insecticide Spirotetramat [60].

Exposure to the same concentrations of Movento^®^ 240SC in mutant *ATM^tefu^* of *D. melanogaster* demonstrates that the production of DSB in the DNA of germarium cells activates DDR mediated by ATM kinase, and both ATM^tefu^ and ATR^mei−29D^ kinases are only activated in the presence of DSB at the highest concentration (37.3 mg/L). Some studies have indicated that ATR may have functional redundancy with ATM even by activating effectors initially phosphorylated by ATM [61,62]. This could support the fact that we observed a significant increase in the expression of γH2AV in the germarium of females of both strains at the highest concentration. A possible contributor to this process could be the induction of oxidative stress that derives from the increased production of ROS that can oxidize bases and inducing single- and double-strand breaks (SSBs and DSBs) [59]. Our results are supported by the data reported in coelomocytes of earthworms (*Esenia fetida*) exposed to 1.25 and 2.5 mg/kg^−1^ of Spirotetramat for 14, 21 and 28 days, in which induction of oxidant stress and lipoperoxidation correlated with DNA fragmentation were determined [14]. An important response to the induction of DNA damage is the activation of cell cycle checkpoints to initiate DNA repair processes. This mechanism is regulated by the activation of the ATR and ATM-dependent Chk1 and Chk2 kinases, respectively [63]. In this study, the exposure to Movento^®^ 240SC in *Chk1^grp^* and *Chk1^grp^*/*Chk2^lok^* mutant strains demonstrates the increase in DNA damage and the deficiency of DNA repair mechanisms in regions 1, 2a and 2b of the germarium, and consequently, induction of cell death evidenced by the absence of DAPI-stained cell nuclei. The significant increase in the expression of γH2AV in *p53^dp53^* mutant females in relation to the Oregon R wild-type strain (*p <* 0.005) only at the highest concentration of 37.3 mg/L of Movento^®^ 240SC, allowed us to infer that, in general, the mechanisms of DNA damage response and repair are mainly regulated by Chk2^lok^, but not dependent on p53^dp53^. Additionally, it supports our hypothesis that this concentration generates many ROS that activate multiple DNA damage response and repair mechanisms. In addition to activating p53, Chk2 can regulate the activation of transcription factors, such as FOXO1 [64]. FOXO1, in a similar way to p53, also regulates processes such as cell proliferation and survival [65,66] as well as responding to the induction of oxidative stress [67]. This could support the absence of cell nuclei observed in the germarium in females of the *Chk1^grp^*/*Chk2^lok^* deficient mutant strain, where, in the absence of Chk2, FOXO1 cannot be activated and consequently regulate cell proliferation and survival, in addition perhaps to responding to oxidative stress induction. Negative regulation of FOXO1 expression has already been reported in female mice ovaries exposed to Movento, in which histological analysis revealed damage to cell membranes, nuclear fragmentation, and cell death of ovarian granulosa cells [68].

In relation to the commercial insecticide Envidor^®^ 240SC, after exposure to concentrations of 12.3, 24.6 and 41.1 mg/L, we determined a significant increase in the percentage of γH2AV expression in the germarium of *ATM^tefu^* and *ATR^mei−29D^* mutant females independently, which could perhaps indicate that Envidor^®^ 240SC acts as a genotoxic agent through the induction of SSBs (single-strand breaks), which consequently lead to the formation of DSBs. ATR is activated when SSBs and stress occur during replication [69]. In response, ATR regulates replisome stability, activation of the origin of replication, and prevents premature mitotic entry [70,71]; failure in any of these may lead to an accumulation of SSBs and aberrant DNA structures (apurinic/apyrimidinic sites) that are difficult to repair and can lead to the formation of DSBs [72], simultaneously activating ATM and ATR [73]. Additionally, we observed that, at a concentration of 24.6 mg/L in both *ATM^tefu^* and *ATR^mei−29D^* mutant strains, cell death is induced, evidenced by the absence of nuclei in region 3, perhaps derived from the induction of oxidative stress and ROS production that could be interfering with the DNA damage response and repair mechanisms, leading to cell death. A recent study reported that Spirodiclofen (the active ingredient of Envidor^®^ 240SC) increases the frequency of micronuclei (MN), chromosomal aberrations (CA) and DNA fragmentation, in addition to decreasing the percentage of the mitotic index in meristem cells of *Allium cepa*, correlated with the induction of oxidative stress and lipid peroxidation. Spirodiclofen administration also caused structural damage, such as cell wall thickening, flattened cell nucleus, cell deformation and necrosis [12]. The activation of Chk1 and Chk2 kinases is a key process in cell cycle arrest and activation of DNA damage response and repair mechanisms [74,75]. The significant increase in the percentage of γH2AV expression in the germarium of *Chk1^grp^* mutant females compared to Oregon R wild-type females (*p <* 0.0001), supports our hypothesis that Envidor^®^ 240SC primarily induces SSBs and that, in this context, lesion repair initially depends on ATR-mediated activation of Chk1. However, SSB formation ultimately leads to DSB formation exceeding Chk1 repair capacity, upon which Chk2 is activated to initiate repair mechanisms and/or cell death [76,77]. This would explain why, in mutant females for both *Chk1^grp^* and *Chk1^grp^*/*Chk2^lok^* kinases, we identified a significant increase in DNA damage, and an absence of cell nuclei in all regions of the germarium, which could also be a consequence of the induction of oxidative stress. With the significant increase in DNA damage induced by the insecticide Envidor^®^ 240SC in *p53^dp53^* mutant females, we define its direct participation in activating DNA damage response and repair mechanisms [78] but not in the activation of processes that lead to cell death, and therefore, perhaps similarly as with Movento^®^ 240SC, there are other effectors downstream of Chk2 that initiate cell death processes, such as BRCA1 and transcription factors such as FOXO1 and E2F1, that also regulate the proliferation and induction of cell death [64,79].

**Figure 7:**
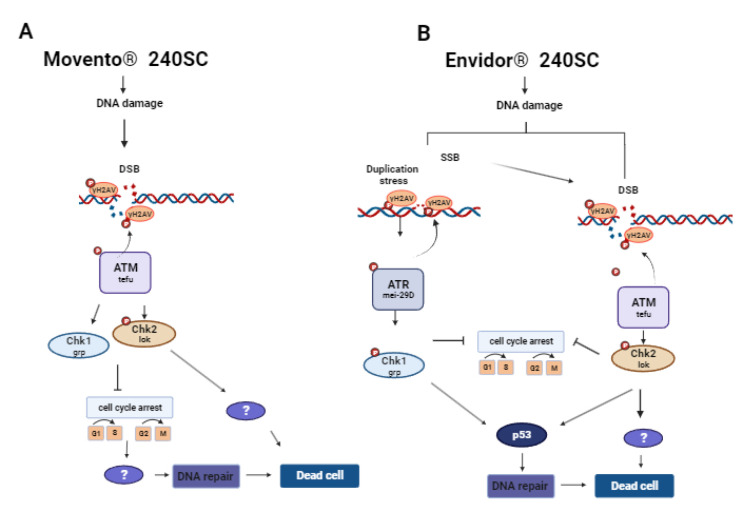
DNA damage response (DDR) mechanism induced by keto-enol insecticides. (**A**) DDR induced by Movento^®^ 240SC. (**B**) DDR induced by Envidor^®^ 240SC.

Based on our results, we hypothesized two possible response mechanisms to DNA damage induced by the keto-enol insecticides Movento^®^ 240SC and Envidor^®^ 240SC (Figure 7A,B).

## 5. Conclusions

Based on our results, we show that the commercial keto-enol insecticides Movento^®^ 240SC and Envidor^®^ 240SC act as genotoxic agents by inducing DSBs, and Envidor^®^ 240SC induces SSBs, in regions 2b and 3 of the *D. melanogaster* germarium, activating two DNA damage response mechanisms (DDR). Movento^®^ 240SC depends on the activation of ATM^tefu^, Chk1^grp^ and Chk2^lok^ kinases, with ATR^mei−29D^ and p53^dp53^ kinases only responding at the highest concentration of 37.3 mg/L. With the Envidor^®^ 240SC insecticide, we determined that the DDR depends on the activation of the ATR^mei−29D^/Chk1^grp^ and ATM^tefu^/Chk2^lok^ kinases, and that the repair mechanisms are dependent on p53^dp53^. The data obtained support our hypothesis that both keto-enol insecticides represent a potential risk for the female reproductive system, affecting the proliferation, maturation, and development of germ cells, which could consequently affect the quality of the oocytes and the fertility rate. However, further studies are still required to fully elucidate the DNA response and repair mechanisms induced by this class of insecticides.

## Figures and Tables

**Table 1:** *Drosophila melanogaster* strains.

Genotype in the Text	Genotype ^1^	Characteristic
Oregon R	+	Wild-type strain competent in all DNA damage response mechanisms.
*ATM^tefu^*	w; tefu e^6^ [b^e^]	Mutant deficient in the protein kinase tefu (*telomere fusion*), homologue of ATM in mammals.
*ATR^mei−29D^*	w; mei-41^29D^/y{UASp41} mei-41^29D^; p{mtα}/+	Mutant deficient in the protein kinase mei-41 (*meiotic-41*), ortholog of ATR in mammals.
*Chk1^grp^/Chk2^lok^*	w/+; grp^209^ lok^30^/grp^Z5170^ lok^30^	Mutant deficient in the protein kinases: grp (*grappes*) and lok (*localized ovarian kinase*), respectively orthologs of Chk1 and Chk2 in mammals.
*Chk1^grp^*	w/+; grp^209^/grp^Z5170^	Mutant deficient in the protein kinase grp (*grapes*), ortholog of Chk1 in mammals.
*p53^dp53^*	y^1^ w^1118^; p53^5A−1−4^	Mutant deficient in the dp53 protein (tumor suppressor), ortholog of p53 in mammals.

^1^ All the strains were kept at 25 ± 2 °C, in vial with standard culture medium based on yeast, agar, sucrose and flour.

## Data Availability

Not applicable.
